# *Trypanosoma cruzi*-infected *Panstrongylus geniculatus* and *Rhodnius robustus* adults invade households in the Tropics of Cochabamba region of Bolivia

**DOI:** 10.1186/s13071-016-1445-1

**Published:** 2016-03-16

**Authors:** Mirko Rojas-Cortez, Maria-Jesus Pinazo, Lineth Garcia, Mery Arteaga, Liliana Uriona, Seyla Gamboa, Carolina Mejía, Daniel Lozano, Joaquim Gascon, Faustino Torrico, Fernando A. Monteiro

**Affiliations:** Fundación CEADES, Cochabamba, Bolivia; ISGlobal, Barcelona and Center for International Health Research (CRESIB), Hospital Clinic – Barcelona University, Barcelona, Spain; Instituto de Investigaciones Médicas (IIBISMED), Facultad de Medicina, Universidad Mayor de San Simón, Cochabamba, Bolivia; Laboratório de Epidemiologia e Sistemática Molecular, Instituto Oswaldo Cruz (Fiocruz), Rio de Janeiro, Brazil

**Keywords:** *Panstrongylus geniculatus*, *Rhodnius robustus*, *Trypanosoma cruzi*, Tropics of Cochabamba, Bolivia

## Abstract

**Background:**

There are hardly any data available on the relationships between the parasite and the vector or regarding potential reservoirs involved in the natural transmission cycle of *Trypanosoma cruzi* in the Tropics of Cochabamba, Bolivia. Local families from communities were responsible for the capture of triatomine specimens, following a strategic methodology based on entomological surveillance with community participation developed by the National Chagas Programme of the Ministry of Health of Bolivia.

**Findings:**

We describe the collection of adult *Panstrongylus geniculatus* and *Rhodnius robustus* naturally infected with *Trypanosoma cruzi* from houses and from the hospital of Villa Tunari municipality. The flagellates found in the digestive tract of *P. geniculatus* belong to genetic lineages or DTUs TcI and TcIII, whereas only lineage DTU TcI was found in *R. robustus*. The detection of these vectors infected with *T. cruzi* reveals the vulnerability of local communities.

**Conclusion:**

The results presented here highlight the risk of Chagas disease transmission in a region previously thought not to be endemic, indicating that the Tropics of Cochabamba should be placed under permanent entomological and epidemiological surveillance.

**Electronic supplementary material:**

The online version of this article (doi:10.1186/s13071-016-1445-1) contains supplementary material, which is available to authorized users.

## Background

Chagas disease transmission to humans occurs when they come in contact with *Trypanosoma cruzi*-infected feces of hematophagous insects of the subfamily Triatominae (Hemiptera: Reduviidae) [1]. Epidemiologically important triatomine species are those with the ability to efficiently colonize rural habitations where they may transmit the *T. cruzi* to local inhabitants. Of the 17 currently recognized triatomine species that occur in Bolivia, only *Triatoma infestans* fulfills the above definition, being responsible for 80 % of vector-borne Chagas disease transmission in endemic areas such as the mesothermal valleys and the Chaco [2].

Other native vectors are also often reported in Bolivia, including *Triatoma sordida* and *Panstrongylus megistus* in the valleys, and *Rhodnius stali*, *R. robustus*, *Panstrongylus rufotuberculatus* and *P. geniculatus* in the Amazon region and Yungas [3–5]. Light-attracted and in search of food sources, these vectors occasionally invade human dwellings, thus presenting the possibility of *T. cruzi* transmission to humans [4].

The Amazon region and the Yungas have not been considered endemic for Chagas disease. However, several studies have documented the presence of *Rhodnius* specimens in palm trees close to rural households both in the Yungas region, La Paz Department, and in the Cochabamba Tropic region [4, 5].

The *Rhodnius robustus* species complex is distributed throughout the Amazon region [6]. These taxa utilise several Amazonian palm tree species as natural habitats, and might show high percentages of natural infection with *T. cruzi* [7].

A preferred habitat of *Rhodnius* spp. are non-cultivated palm trees with dense foliage as they provide shelter for a wide variety of mammals and birds, which serve as food sources for the insects [8, 9].

*Panstrongylus geniculatus*, on the other hand, has a very wide geographical distribution, from Southern Mexico to Northern Argentina [10]. In Bolivia, this vector has been identified in the Yungas region, the eastern plains, and the Bolivian Chaco [4]. Despite its occasional entry into homes attracted by light, this species does not colonize human dwellings, which is an important factor limiting its potential as an efficient vector of Chagas disease [11].

In the natural habitats, *P. geniculatus* can be found in caves which provide shelter to armadillos, anteaters, marsupials, bats and rodents. They can also be found in palm trees, under trunks and bark and in bromeliad plants [12, 13].

An increasing number of food-borne Chagas disease outbreaks have been caused by different sylvatic vectors [14]. Most cases of Chagas disease transmission *per os* have occurred in Brazil, but they have also been reported less frequently in countries such as Colombia, Argentina, Venezuela, Ecuador and Bolivia [15].

More than 180 domestic, synanthropic and wild species of mammals, especially nest-building rodents, opossums and primates, are likely to be infected with *T. cruzi*.

*Trypanosoma cruzi* is now classified into seven discrete typing units (DTUs): *T. cruzi* I (TcI) to *T. cruzi* VI (TcVI) and Tcbat. TcI has the widest distribution ranging from southern USA to northern Argentina and Chile; TcII is found predominantly in the southern and central regions of South America; TcIII and TcIV have both been detected from western Venezuela to the Argentinian Chaco; TcV and TcVI occur in southern and central South America; and Tcbat is reported from the Amazonian rainforest to urban areas of the central, northeast and southeast Brazil [16–18].

The Tropics of Cochabamba region is technically referred to as a *bosque de uso múltiple*. Its population belongs predominantly to the Quechua ethnic group and the economy relies basically on the harvesting of coca leaves and tropical fruits, with fish farming and tourism playing secondary roles. This geographical area is not considered epidemiologically important because it is located outside the endemic region (i.e. where *T. infestans* occurs).

The Chagas disease epidemiological scenario in the region has gone through important changes. This region has experienced intense migration of people coming from highly endemic Andean regions, leading to the development of many new settlements. These migration flows were stronger in the 50s due to the Bolivian Agrarian Reform and in the 80s due to a combination of economic hardship and adverse weather conditions caused by climate change [19].

The main goal of this study was to identify, based on a community-oriented surveillance program, the sylvatic Chagas disease vectors that most often invade households in the Tropic of Cochabamba region, in Bolivia, and determine their natural infection rates and identify the strain of *T. cruzi*.

## Methods

Triatomine specimens were obtained from local residents of communities in the Tropics of Cochabamba between August 2012 and January 2014, in the framework of action of the Villa Tunari Center for Comprehensive Chagas Disease Care (VTCCCDC), a collaborative project between the *Colectivo de Estudios Aplicados y Desarrollo Social* of Bolivia (CEADES) and the Institute for Global Health (ISGlobal), Barcelona, Spain. Residents carried out the capture of triatomine species according to a strategic methodology based on entomological surveillance with community participation developed by the National Chagas Program (Ministry of Health, Bolivia). Members of each household received an educational folder with instructions on how to search for triatomines in their homes (Additional file 1: Fig. S1). After notification by the residents, house inspection for insects was carried out by the man-hour method.

The entomological surveillance folder was designed to be used in tropical non-endemic regions where triatomines are not known to colonize houses. It provides basic information on Chagas disease, a schematic representation of all community players that take part in the initiative, and an entry for family data. Inside there are two figures illustrating both the peridomestic and intradomestic areas with directions on how to clean and maintain each area in order to prevent triatomine colonization. Earlier studies had identified the presence of *Rhodnius robustus* in palm trees of the Tropics of Cochabamba region [MR-C, personal communication]. Thus, the developmental cycle for this species was included in the folder to facilitate vector identification by the participants. Information on how to avoid oral Chagas disease transmission is also provided (Additional file 1: Fig. S1).

Collected triatomines were delivered to the VTCCCDC, where they were morphologically identified according to the taxonomic keys of Lent & Wygodzinsky [3], and stored in the laboratory for further use.

For the microscopic observation, drops of feces extracted from triatomines were mixed with phosphate-buffered saline solution and viewed at a magnification of 400×. A sample was considered positive whenever flagellate parasites were detected during a 5 minute examination under the microscope. The confirmation of *T. cruzi* infection was done based on the molecular method described below.

To confirm insect infection by *T. cruzi* and to characterize the strains to which they belong, DNA was obtained from the insects’ digestive tracts using a DNA extraction kit DNAzol® reagent according to the manufacturer instructions. A first PCR amplification was performed according to [20]. Amplification was done in 25 μl reaction volume containing 1.5 mM MgCl_2_, 50 μM of each nucleotide, 10 pmol of each primer, 1.25 units of Taq DNA polymerase (Bioline UK) and 20 ng of DNA template. PCR products were analyzed in 3.0 % agarose gels stained with Sybersafe (Invitrogen, Massachussets, USA). This approach discriminates three groups of DTUs: a 200 bp PCR product for TcI; a 205 bp product for TcII, TcV, and TcVI; and a 150 bp product for TcIII and TcIV.

To separate between the TcIII and TcIV DTUs we performed a second PCR amplification, using oligonucleotides to amplify the D7 divergent domain of the LSU rDNA [21]. Amplification was achieved in a final reaction volume of 20 μl containing 2.0 mM MgCl_2_, 200 μM deoxynucleotide solution mix (New England Biolabs, UK), 20 pM of each primer, 1.0 unit of *Taq* DNA polymerase, and 20 ng of DNA template. After amplification, the 125 bp fragments were identified as DTU TcIII, and the 100 bp fragments as DTU TcIV on 3.0 % agarose gels.

## Results and Discussion

Educational folders were distributed to the local population at the Villa Tunari Center for Comprehensive Chagas Disease Care between August 2012 and January 2014. Of a total of 1,427 folders handed-out, 988 subjects (i.e. households; 69 %) participated in the study. Twenty-one households were positive for the presence of two triatomine species: *P. geniculatus* and *R. robustus* (Table 1).

**Table 1:** Triatomines and other Hemiptera collected in houses through the entomological surveillance with community participation in the tropics of Cochabamba, Bolivia

Municipality	No. of folders distributed	No. of folders filled-out (%)	No. of households positive for triatomines (%)			
				*R. robustus*	*P. geniculatus*	Phytophagous or predator hemipterans
Villa Tunari	732	487 (66)	13 (2.7)	2	11	25
Shinahota	200	140 (70)	4 (2.9)	2	2	14
Chimore	181	136 (75)	4 (3.0)	1	3	12
Entre Rios	76	49 (65)	0 (0.0)	-	-	6
Puerto Villarroel	238	176 (74)	0 (0.0)	-	-	4
Total	1427	988 (69)	21 (2.1)	5	16	61

It is worth mentioning that 61 households collected/reported other (non-triatomine) insects showing their commitment and willingness to participate in the entomological surveillance study.

The capture of *P. geniculatus* and *R. robustus* was carried out in different communities from Villa Tunari, Shinahota and Chimore. Only adult insects were collected. From 19 reports, ten indicated that the triatomines were captured inside houses, mainly in bedrooms, and two in a peridomestic area (16 for *P. geniculatus* and five for *R. robustus*). Four *P. geniculatus* reports referred to the locations inside the Villa Tunari Hospital. These insects were captured at night when they were presumably attracted by indoor lighting. Colonies of *P. geniculatus* and *R. robustus* were not detected neither in inspected houses nor in the hospital (Table 2).

**Table 2:** Geographical location of Panstrongylus geniculatus and Rhodnius robustus collected by community members in the Tropics of Cochabamba region, and determination of Trypanosoma cruzi DTUs of infected specimens

N°	Collection site Municipality, Village	Captured intra/peri^a^	Latitude (S)	Longitude (W)	Altitude (m.a.s.l.)	Triatomine species (♀/♂)	Trypanosomatid infection by microscopy	*T. cruzi* DTU determined by PCR
1	Shinaota, Shinaota	Peri	16°59´41.6˝	65°10´51.7˝	249	*P. geniculatus* ♀	-	negative
2	Shinaota, Ibuelo	Intra	16°57´28.8˝	65°16´51.3˝	242	*P. geniculatus* ♀	ND^b^	TcIII
3	Chimoré, Chimoré	Intra	16°59´22.0˝	65°09´21.1˝	248	*P. geniculatus* ♀	+	TcIII
4	Chimoré, Senda III	Intra	16°59´44.0˝	65°11´30.0˝	251	*P. geniculatus* ♂	ND^b^	TcI
5	Chimoré, Senda III	Intra	16°59´33.8˝	65°10´55.7˝	247	*P. geniculatus* ♂	ND^b^	TcI
6	Villa Tunari, General Román	Intra	16°57´22.5˝	65°23´54.7˝	278	*P. geniculatus* ♀	ND^b^	negative
7	Villa Tunari, San Miguel	Intra	16°54´07.9˝	65°22´06.6˝	250	*P. geniculatus* ♀	+	TcI
8	Villa Tunari, SendaBahuer	Intra	16°52´38.6˝	65°23´05.8˝	239	*P. geniculatus* ♂	ND^b^	TcI
9	Villa Tunari, Isinuta	Intra	16°43´44.9˝	65°38´31.1˝	237	*P. geniculatus* ♂	ND^b^	negative
10	Villa Tunari, Capihuara	Intra	16°59´29.3˝	65°37´35.8˝	473	*P. geniculatus* ♂	ND^b^	TcI
11	Villa Tunari, Capihuara	Intra	16°59´28.8˝	65°37´37.9˝	471	*P. geniculatus* ♀	ND^b^	negative
12	Villa Tunari, Cristal Mayu	Intra	16°59´23.4˝	65°37´13.9˝	473	*P. geniculatus* ♀	+	TcI
13	Villa Tunari, San Francisco de Asis Hospital	Intra	16°58´43.0˝	63°25´30.7˝	313	*P. geniculatus* ♂	+	TcI
14	Villa Tunari, San Francisco de Asis Hospital	Intra	16°58´42.8˝	63°25´30.2˝	312	*P. genicultaus* ♀	ND^b^	TcIII
15	Villa Tunari, San Francisco de Asis Hospital	Intra	16°58´42.8˝	63°25´30.2˝	312	*P. genicultaus* ♀	ND^b^	negative
16	Villa Tunari, San Francisco de Asis Hospital	Intra	16°58´42.8˝	63°25´30.2˝	312	*P. genicultaus* ♂	ND^b^	negative
17	Villa Tunari, Todos Santos	Intra	16°56´35.9˝	65°17´34.9˝	245	*R. robustus* ♀	-	negative
18	Villa Tunari, Villa Fernandez	Intra	16°57´28.6˝	65°16´48.1˝	245	*R. robustus* ♂	-	negative
19	Shinaota, San Luis	Peri	16°57´33.0˝	65°17´06.0˝	242	*R. robustus* ♀	ND^b^	negative
20	Shinaota, Ibuelo	Intra	16°59´10.9˝	65°20´39.3˝	272	*R. robustus* ♂	ND^b^	TcI
21	Chimore, Puente Roto	Intra	16°59´46.1˝	65°04´41.2˝	239	*R. robustus* ♂	ND^b^	TcI

^a^ Intra/peri: intradomicile/peridomicile; ^b^ Not determined because specimens were too dry

The numbers of *P. geniculatus* collected from inside houses is somewhat unexpected, perhaps because little attention has been given to the biology and distribution of this particular species in the country. On the other hand, *R. robustus* has been known to occur in the Alto Beni region of Bolivia since 2001 [22].

Ten out of the 12 specimens positive for *T. cruzi* infection were from houses and two were from the Villa Tunari Hospital. Molecular identification methods confirmed that the flagellates found in the digestive tracts of *P. geniculatus* and *R. robustus* belong to DTUs TcI and TcIII for the former, and TcI for the latter (Table 2).

In the wild, *P. geniculatus* has usually been described as a vector of TcIII, associated with armadillos throughout the Americas, but it has also been found to be infected with TcI, TcII, and TcIV in Venezuela, Colombia, and Brazil [23–25]. Among TcI isolated from lowland Bolivia, Poisson-distributed allele frequencies point to large, stable effective population sizes [26]. In this specific region of Bolivia, however, there is little available data about the relationship between parasite DTU and this particular vector species. In addition, information about sylvatic reservoirs that would help clarify the natural transmission cycle is also lacking.

The VTCCCDC has seen an increase in the number of adult vectors reported by local inhabitants. Of the 17 families that reported the presence of *P. geniculatus* and *R. robustus* in their houses, 15 were migrants from endemic areas of Bolivia. Each family had at least one member with positive serology for *T. cruzi* infection. One of the two indigenous families found to have the vector in their houses, had a family member with positive serology for *T. cruzi* infection (unpublished data).

In October 2010, the National Chagas Program reported an outbreak of acute Chagas disease in Guayaramerín, Beni region, in the Bolivian Amazon, which placed health officials on alert. Fourteen cases of acute Chagas disease were confirmed by laboratory diagnosis at local and national reference centers. All parasites were molecularly identified as belonging to DTU TcIV, leading to the suggestion that all analyzed patients were exposed to the same source of infection [27].

The findings of Coura et al. [28] in Brazil, Carrasco et al. [29] in Venezuela and Santalla et al. [27] in Bolivia, draw attention to the increased seroprevalence of *T. cruzi* infection in regions hitherto not considered endemic for Chagas disease.

The increased migration and settlement (colonization) of infected people from endemic areas, coupled with the evidence of house invasion by sylvatic triatomine species, has motivated health officials to take action in such tropical regions.

The peoples’ willingness to participate in the study exceeded our expectations as most of them (colonizers) were familiar with *T. infestans* but had no prior knowledge of sylvatic bugs that could transmit *T. cruzi* in tropical regions. This indicates that our public awareness approach using a folder as a surveillance method was effective.

We suggest that environmental changes could be causing an impact on the ecology and behavior of sylvatic vectors. Deforestation, house building, availability of electricity, added to a new blood source, would be favoring household invasion by such vectors. Unfortunately, we have no information regarding the daily behavior and practices of the colonizing families that may be increasing the risk of domiciliation by sylvatic insects.

## Conclusion

The detection of *P. geniculatus* and *R. robustus* infected by *T. cruzi* in the urban areas of Cochabamba studied, indicates the vulnerability of the local human population to *T. cruzi* infection. Therefore, the Tropics of Cochabamba, previously thought not to be endemic, should receive attention by health officials and, if necessary, be placed under permanent entomological and epidemiological surveillance.

## Additional file

Additional file 1: Fig. S1. Entomological surveillance folder designed to be used in tropical non-endemic regions where triatomines do not colonize houses. (DOC 2007 kb)
