# Supplementary material for: Trypanosoma cruzi-infected Panstrongylus geniculatus and Rhodnius robustus adults invade households in the Tropics of Cochabamba region of Bolivia
Source: Parasit Vectors. 2016 Mar 16;9:158. doi: 10.1186/s13071-016-1445-1 (PMC4794895; doi:10.1186/s13071-016-1445-1)

**Figure legend**

Figure S1. Entomological surveillance folder designed to be used in tropical non-endemic regions where triatomines do not colonize houses.

The outside of the folder


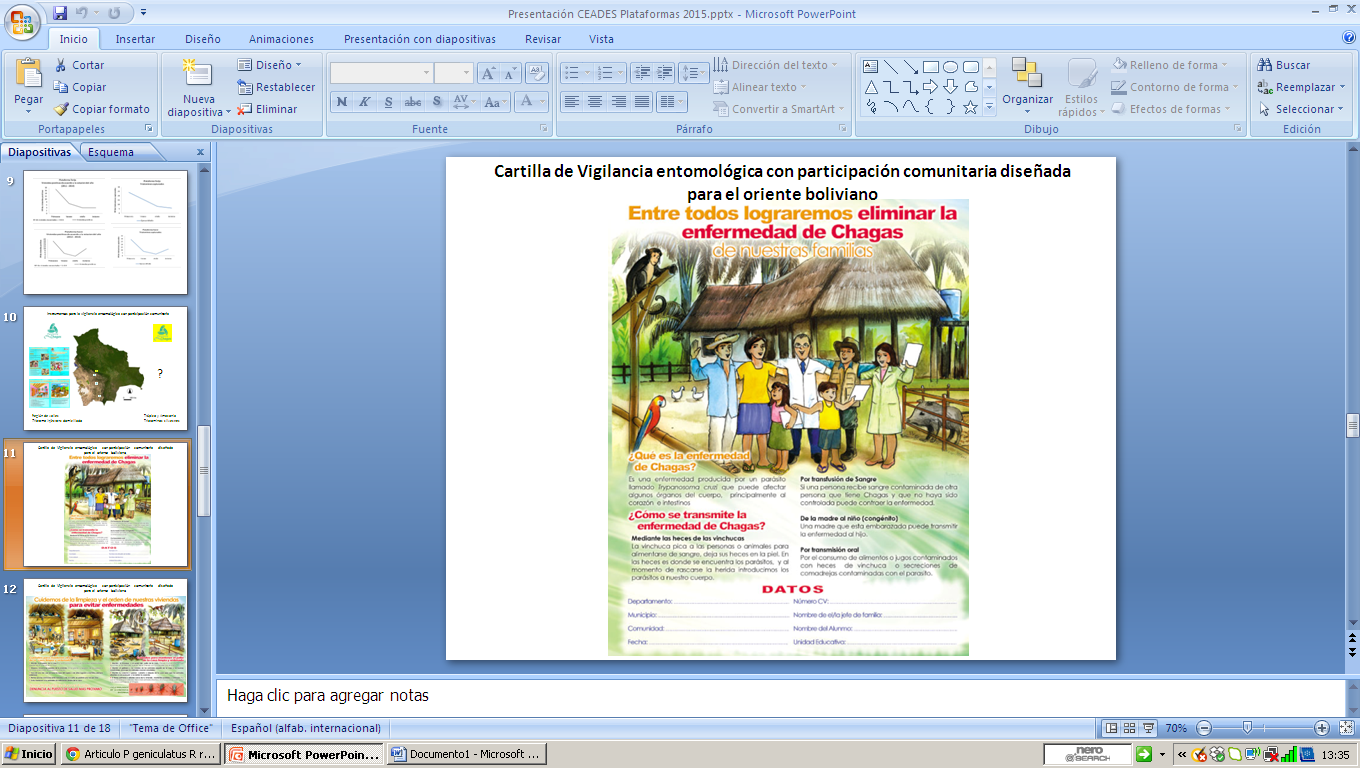

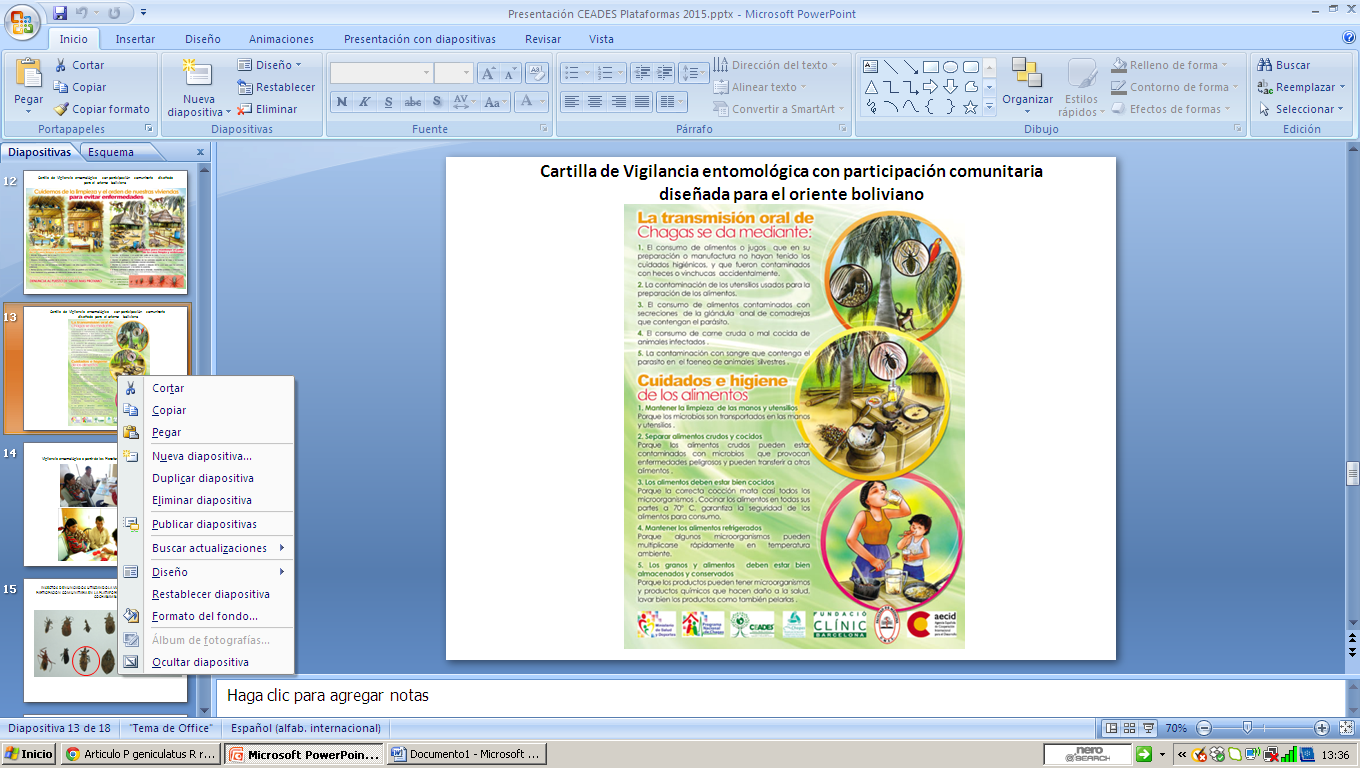


The inside of the folder


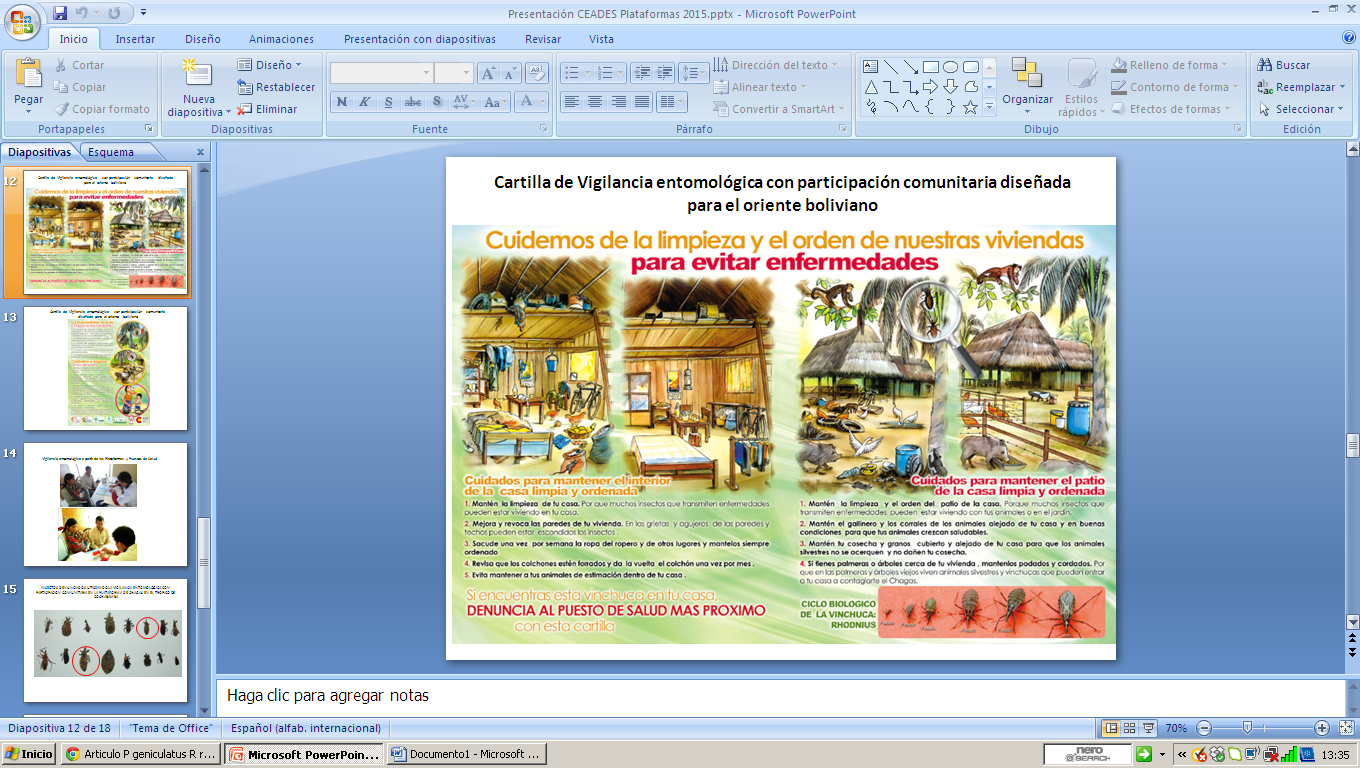

Supplement: Additional file 1: Fig. S1. — Entomological surveillance folder designed to be used in tropical non-endemic regions where triatomines do not colonize houses. (DOC 2007 kb) [file 13071_2016_1445_MOESM1_ESM.doc]
